# Green Synthesis of Molybdenum Nanoparticles From Solanum xanthocarpum and Evaluation of Their Antimicrobial and Antioxidant Activity Against Multidrug-Resistant Wound Isolates

**DOI:** 10.7759/cureus.56760

**Published:** 2024-03-23

**Authors:** Priyam Bharathidasan, Muthuvel Surya, P Geetha Sravanthy, Muthupandian Saravanan

**Affiliations:** 1 Department of Pharmacology, Saveetha Dental College and Hospitals, Saveetha Institute of Medical and Technical Sciences, Chennai, IND

**Keywords:** multidrug resistance, antioxidant activity, antimicrobial, molybdenum nanoparticles, green synthesis

## Abstract

Introduction: In recent years, antimicrobial drug resistance has emerged as a serious global public health concern, according to the World Health Organization data. The emergence of pathogens resistant to multiple drugs has been linked to an increase in morbidity and mortality from microbial infections. The study's main goal is to explore the efficacy of using *Solanum xanthocarpum* in the green synthesis of molybdenum nanoparticles (Mo NPs) for antibacterial and antioxidant properties.

Methods: An eco-friendly method of synthesizing Mo NPs was accomplished using an aqueous extract of *Solanum xanthocarpum*. Characterization of the synthesized nanoparticles was done by UV-visible spectroscopy (UV-Vis), Fourier-transform infrared spectroscopy (FT-IR), X-ray diffraction (XRD), scanning electron microscope (SEM), and energy-dispersive X-ray spectroscopy (EDX). After that, antibacterial and antioxidant activity was further evaluated.

Results: The UV-visible spectrophotometer analysis confirmed the presence of synthesized Mo NPs showing a peak around 320 nm. The presence of functional compounds like C-CI, C-H, C=C, and O=C=O was confirmed by FT-IR spectrum analysis. The positions of diffraction peaks in Mo NP patterns were identified using XRD analysis; they were more crystalline (82.7%) and less amorphous (17.3%). The presence of the elements molybdenum (Mo), carbon (C), and oxygen (O) was confirmed by the EDX spectrum and irregular shapes shown in the SEM images. Further, the antimicrobial study results showed the formation of an inhibition zone against 27 mm for *Klebsiella pneumoniae*, 24 mm for *Pseudomonas aeruginosa*, 22 mm for *Staphylococcus aureus*, and 24 mm for *Enterococcus faecalis*, respectively, at a high concentration 80 μg/ml of Mo NPs. The maximum antioxidant activity at 100 μg/ml was 73.49%, compared to the standard ascorbic acid (74.25%). Additionally, the moderate activity at 60 μg/ml was 53.21%, compared to the standard (56.5%), and the minimal activity at 20 μg/ml was 30.21%, compared to the standard (36.89%).

Conclusion: The environmentally friendly synthesized Mo NPs from *Solanum xanthocarpum* exhibited antioxidant activity. Furthermore, the findings show that Mo NPs mediated by *Solanum xanthocarpum* can inhibit antibiotic-resistant bacteria, especially methicillin-resistant *Staphylococcus aureus*, *Klebsiella pneumoniae*, *Pseudomonas aeruginosa*, and *Enterococcus faecalis*. In order to understand further how nanoparticles work against bacteria that are resistant to many drugs, additional research and clinical studies would be needed.

## Introduction

Bacteria are considered multidrug-resistant (MDR) if they are capable of resisting many antimicrobial drugs that come from different chemical classes or subclasses at a single time with separate pathways [1]. The results of an in vitro antimicrobial susceptibility test are one of the techniques that many authors and authorities use to identify organisms as MDR. Antimicrobial drug resistance can be acquired by an organism and encoded chromatically, or it can develop from mutation or the horizontal gene transfer of resistance genes [2]. MDR is a major concern for physicians, patients, and pharmaceutical corporations in hospitals as well as in the community [3]. The extensive usage of antibiotics and the length of time the drugs have been on the market have resulted in major problems with the evolution of resistant microbes [4]. In Atlanta, Georgia (USA), research on multidrug resistance in gram-negative bacteria that cause healthcare-associated illnesses revealed that 15% of *Klebsiella pneumoniae* and 10% of *Pseudomonas aeruginosa* were resistant to three different antibiotic classes. Around 60% of the isolates of *Acinetobacter baumannii *were resistant to three or more antibiotic classes, which is a far higher percentage [5].

The potential uses of plant extracts as capping, reducing, and regulating agents in the synthesized nanoparticles have gained a lot of attention in recent years. Additionally, therapeutic herbs are frequently used to synthesize nanoparticles [6]. In various regions of India, *Solanum xanthocarpum* grows as a wild annual herb. The common names for it are Bhatkatiya or Kantakari. Berries are characterized by an expanded calyx around a yellow or green stripe [7]. The plant *Solanum xanthocarpum* contains steroidal alkaloids such as solamargine, solacarpidine, and solacarpine. Caffeic acid, triterpenes (cycloartanol and cycloartenol), coumarins (aesculetin and aesculin), and steroids (carpesterol, diosgenin, campesterol, and daucosterol) are other components [8]. *Solanum xanthocarpum *fruit primarily consists of flavonoids, especially apigenin and quercitrin glycosides. This versatile herb has been linked to numerous medical benefits for its various sections [9]. For instance, as an expectorant, the root is used in traditional medicine to treat chest pain, asthma, coughing, and wound healing. Fruits are delicious, contain anthelmintic qualities, and are used as treatments for a variety of disorders. The antioxidant and antihyperglycemic activities of *Solanum xanthocarpum* leaf extracts were evaluated in diabetic rats given alloxan in a recent study [10]. Plant extracts are very effective at reducing metal salts and forming metallic nanoparticles due to their great reducing ability and antioxidant activity [11].

Numerous research have examined the antibacterial properties of metals such as silver (Ag), copper (Cu), and gold (Au) as well as metal oxides like zinc oxide (ZnO), magnesium oxide (MgO), and copper oxide (CuO). Molybdenum trioxide (MoO_3_), a biofunctional compound with antibacterial and anticancer features, has emerged recently [12]. Among all the nanoparticles utilized in medicine, MoO_3_ nanoparticles are the most harmless. They also have high antibacterial properties against a range of bacterial species, including those that cause hospital-acquired diseases, and produce an acidic pH [13]. Due to their antibacterial action, which includes oxidative stress or disruption of membranes that result in bacterial cell wall rupture and finally cell death, MoO_3_ NPs provide a possible alternative [14]. MoO_3_ NPs have strong antibacterial and pro-antigenic properties, which make them very useful for wound healing applications [15]. The current study demonstrated the environmentally friendly synthesis of molybdenum nanoparticles (Mo NPs) from *Solanum xanthocarpum* and assessed their antimicrobial and antioxidant efficacy against isolates from MDR wounds.

## Materials and methods

Materials

Metal oxide MoO_3_ was purchased from Sisco Research Laboratories Pvt. Ltd. HiMedia (Mumbai, India) provided Mueller-Hinton agar (MHA) and 2,2-diphenyl-1-picryhydrazyl (DPPH). The bacterial strains were obtained from the Department of Microbiology at Saveetha Medical College.

Sampling

We collected leaves of *Solanum xanthocarpum* from Poonamallee in Chennai, Tamil Nadu. The taxonomic identity of the sample was confirmed by Dr. N. Siva, an Assistant Professor in the botany department of Raja Doraisingam Government Arts College in Sivagangai, Tamil Nadu.

Extraction

Following rinsing thrice in distilled water, the *Solanum* *xanthocarpum* leaves were allowed to air dry and the sample was ground to a powder using a grinder mixture. After mixing 10 g of powdered *Solanum* *xanthocarpum* with 200 ml of distilled water, the mixture was autoclaved for sterilization. The mixture was then filtered using Whatman No. 1 filter paper (Whatman Plc, Maidstone, UK), and the extract was kept for later use at 4°C.

Biosynthesis of Mo NPs 

The process for biosynthesizing nanoparticles was as follows: Using the titration method, an aqueous extract of *Solanum*​*xanthocarpum *was poured into a conical flask, and 50 mM of MoO_3_ was added to a burette. The burette solution was added dropwise to the aqueous extract. The mixture solution was incubated for the whole night in an orbital shaker. After incubation, It was noted that the color changed from dark to light brown. In addition, the mixture was centrifuged at 4500 rpm for 30 minutes. After that the pellet was removed and the supernatant was washed with distilled water and re-centrifuged at 4500 rpm for 30 minutes. The pellet was then collected and kept for 24 hours at 60°C in a hot air oven. Furthermore, it was kept for future research at room temperature in an airtight container.

Characterization of Mo NPs 

The efficacy, biodistribution, and mechanism of the nanoparticles are influenced by their physicochemical properties. Characterizing the Mo NPs mediated by *Solanum* *xanthocarpum* is necessary for assessing the functional features of the synthesized nanoparticles by their use in various analytical techniques. For example, 1 cm quartz cuvettes were used in UV-visible spectroscopy (Thermo Scientific Evolution 600) to create optically characterized nanoparticles in the 200-1000 nm ranges. Fourier-transform infrared (FT-IR) spectrum was used to identify the functional groups of the synthesis of *Solanum xanthocarpum* using Mo NPs with a Bruker FT-IR spectrophotometer (Bruker, Billerica, USA) in the 4000-500 cm^-1^ range. The phytochemical properties of the crystal lattice are represented by the number of diffraction peaks that arise from X-ray radiation reflecting off the particles. X-ray diffraction (XRD) is an analytical method used to determine isomorphous substitution, evaluate particle size, resolve different molecules, and qualitatively identify active compounds. A scanning electron microscope (SEM) (JSM-7001F, JEOL, Tokyo, Japan) with an accelerating voltage of 20 keV was used to examine the surface morphology of the synthesized *Solanum​​​​​​ xanthocarpum* mediated Mo NPs. The elements included in the Mo NPs were examined using energy-dispersive X-ray spectroscopy (EDX) (JSM-7001F, JEOL, Tokyo, Japan).

Antibacterial activity

Four bacterial strains were tested using the inhibitory zone of *Solanum​​​​​​ xanthocarpum *mediated Mo NPs such as *Enterococcus faecalis, Pseudomonas aeruginosa,* methicillin-resistant S*taphylococcus aureus,* and *Klebsiella pneumoniae.* The antibacterial activity was assessed using the well diffusion method. For 18 hours at room temperature, the bacterial strains were grown in Mueller-Hinton (MH) broth, with the turbidity level adjusted to meet the McFarland standards of 0.5. After preparing the Mueller-Hinton agar (MHA) plates and autoclave, the medium was dissolved with 300 ml of sterile distilled water. The strains were then swabbed into the plate. The sterile tips were used to puncture the well, which was then filled with various concentrations of *Solanum​ xanthocarpum* mediated Mo NPs (20, 40, 60, and 80 μg/ml) along with streptomycin as a positive control. After a 24-hour incubation period, the inhibitory zone was measured on the plates.

Antioxidant activity

The antioxidant property of Mo NPs from *Solanum xanthocarpum* was studied utilizing a DPPH radical scavenging experiment. The process was run on a microtiter plate with 96 wells. Each well should contain a DPPH solution. Following that, Mo NPs were added to each well at different concentrations (20-100 μg/ml), with a blank well left empty. Ascorbic acid (20-100 μg/ml) was used in the preparation of the standard. The plate was incubated for 30 minutes in a dark environment. The absorbance was recorded using a microplate reader at 517 nm in wavelength.

## Results

Synthesis of Mo NPs from *Solanum xanthocarpum*

**Figure 1:**
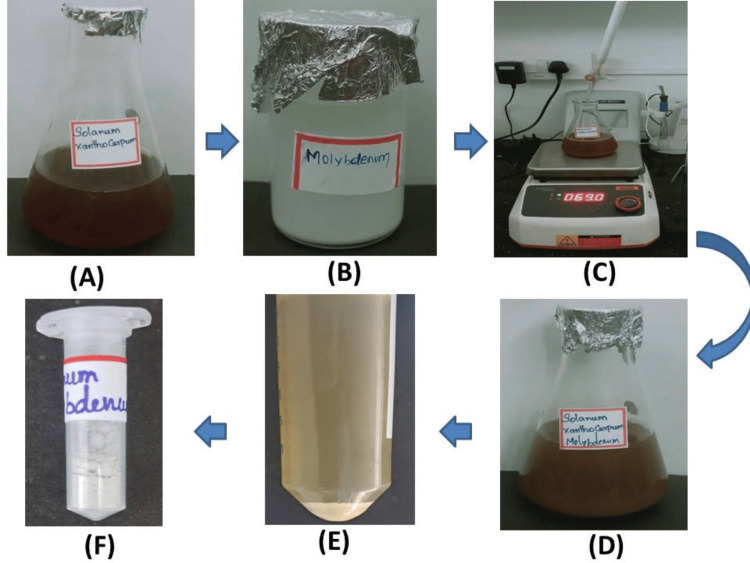
Overview of synthesis of Mo NPs using Solanum xanthocarpum (A) 150 ml aqueous extract of Solanum xanthocarpum; (B) 25 mM of molybdenum trioxide (MoO_3_); (C) Titration process; (D) Synthesized Mo NPs; (E) Centrifugation process; (F) The powder form of Mo NPs Mo NPs: Molybdenum nanoparticles

The molybdenum trioxide was biosynthesized using an aqueous extract of *Solanum xanthocarpum*. The synthesis of Mo NPs using *Solanum xanthocarpum*, as shown in Figure 1, is indicated by the dark to light brown color of the solution.

Characterization of synthesized Mo NPs

UV-Vis Spectral Analysis

**Figure 2:**
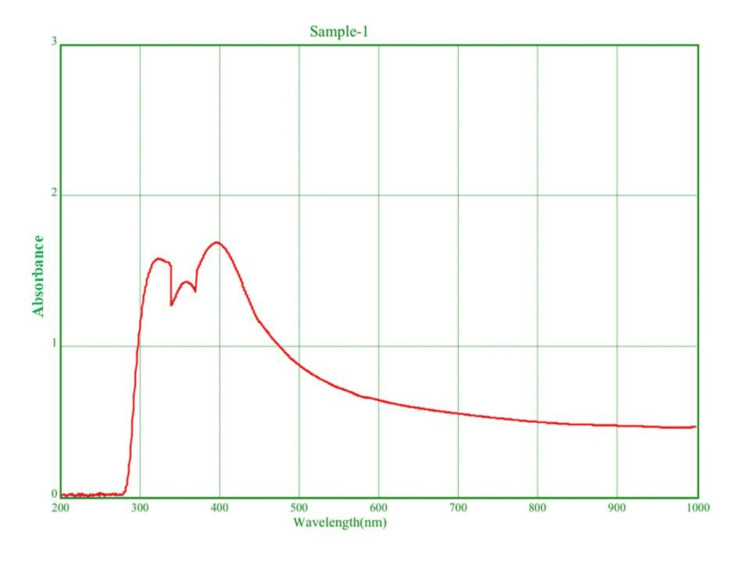
UV-Vis absorption spectra of Mo NPs that were synthesized at 320 nm using Solanum xanthocarpum Mo NPs: Molybdenum nanoparticles

UV-visible spectroscopy was used to determine the wavelength of the plasma resonance peak level of absorbance, and 320 nm was identified as the peak level of absorbance, as shown in Figure 2. Therefore, it was determined that Mo NPs were present.

FT-IR Spectroscopy

**Figure 3:**
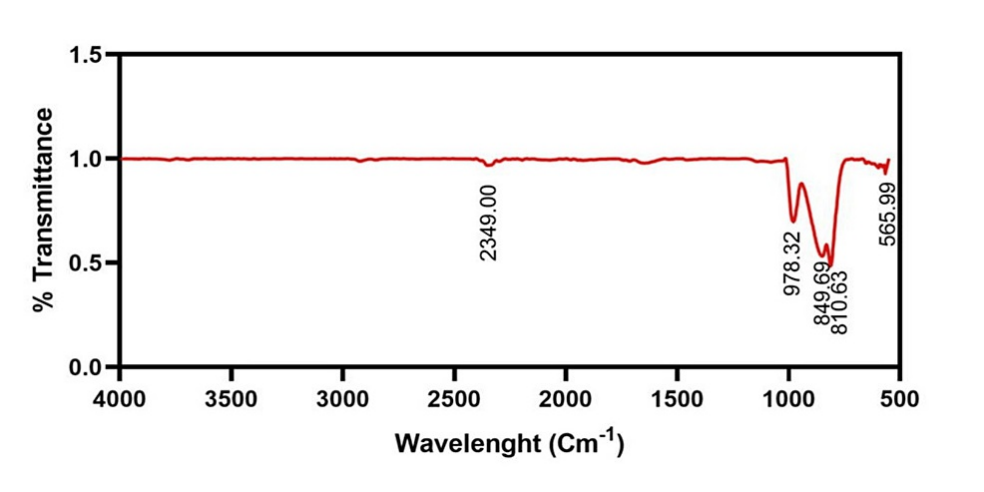
FT-IR spectra of synthesized Mo NPs using Solanum xanthocarpum with more than four functional groups between the 500-4000 cm-1 range FT-IR: Fourier-transform infrared; Mo NPs: Molybdenum nanoparticles

Using the Bruker FT-IR spectrophotometer, the functional groups of the Mo NPs that were synthesized using *Solanum xanthocarpum* were identified. In the 500-4000 cm^-1^ wavelength range, the study revealed that the synthesized *Solanum xanthocarpum* mediated Mo NPs had more than four functional groups. The following values describe the functional groups and chemical bonds: 565.99 cm^-1^, 810.63 cm^-1^, 849.69 cm^-1^, 978.32 cm^-1^, and 2349.00 cm^-1^; these are the most significant values. Functional groups are depicted in Figure 3 and contain C-CI, C-H, 0=C=O, and C=C groups, respectively.

XRD Spectroscopy

**Figure 4:**
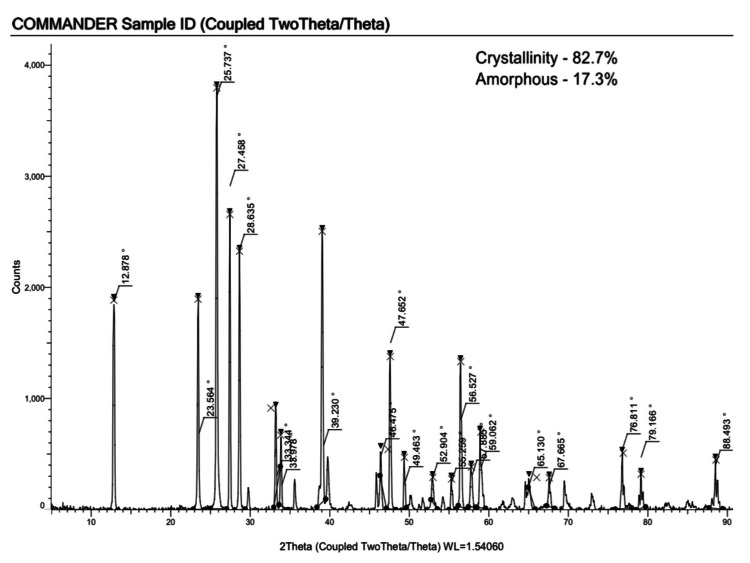
XRD spectra of synthesized Mo NPs using Solanum xanthocarpum with crystalline and amorphous characteristics XRD: X-ray diffraction; Mo NPs: Molybdenum nanoparticles

The XRD method was used to determine the crystalline and amorphous nature of the identified nanoparticles. As a result of our study, synthesized *Solanum xanthocarpum* mediated Mo NPs have more crystalline (82.7%) and less amorphous (17.3%) properties (Figure 4). We therefore synthesized Mo NPs mediated by *Solanum xanthocarpum* with a highly stable crystalline structure.

SEM Analysis

**Figure 5:**
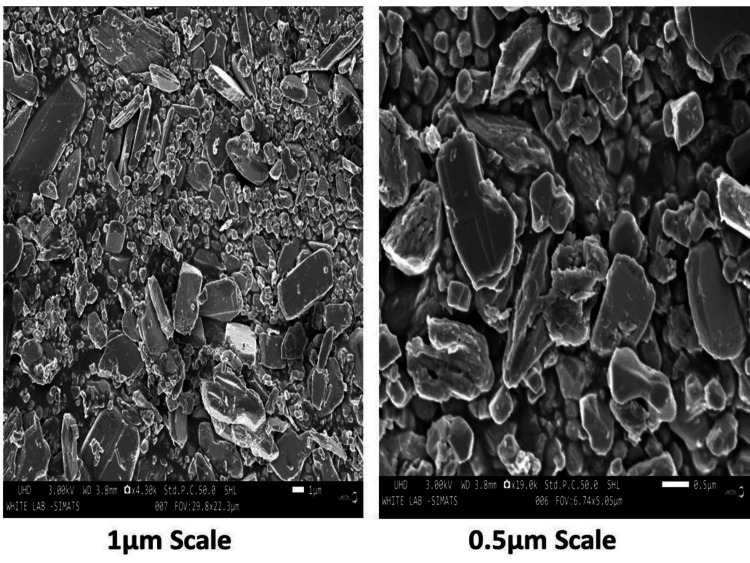
Scanning electron microscopic images of synthesized Mo NPs using Solanum xanthocarpum at 200-300 nm diameters of different magnifications Mo NPs: Molybdenum nanoparticles

The synthetic Mo NPs using *Solanum xanthocarpum* had an irregular shape, shown by two different SEM magnifications (0.5 μm and 1 μm). The presence of the synthesized Mo NPs made from *Solanum xanthocarpum* was confirmed by the average 200-300 nm diameter of the agglomerated nanoparticles (Figure 5).

EDX Analysis

**Figure 6:**
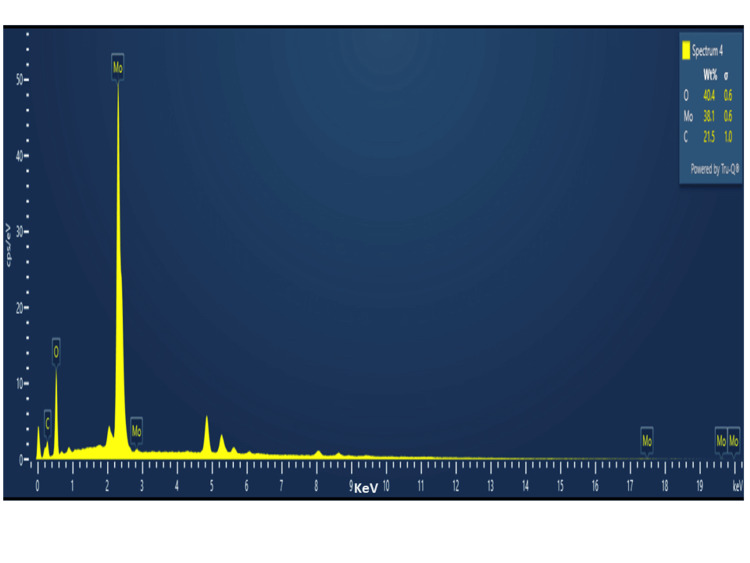
EDX analysis of synthesized Mo NPs using Solanum xanthocarpum with various elemental compositions EDX: Energy-dispersive X-ray spectroscopy; Mo NPs: Molybdenum nanoparticles

Using EDX analysis, the components of *Solanum xanthocarpum *mediated Mo NPs were identified. The molybdenum (Mo), oxygen (O), and carbon (C) signals were found at 38.1, 40.4, and 21.5 keV, respectively, according to the EDX spectra. Mo's surface anchoring is confirmed by this (Figure 6).

Antibacterial efficacy of synthesized Mo NPs

**Figure 7:**
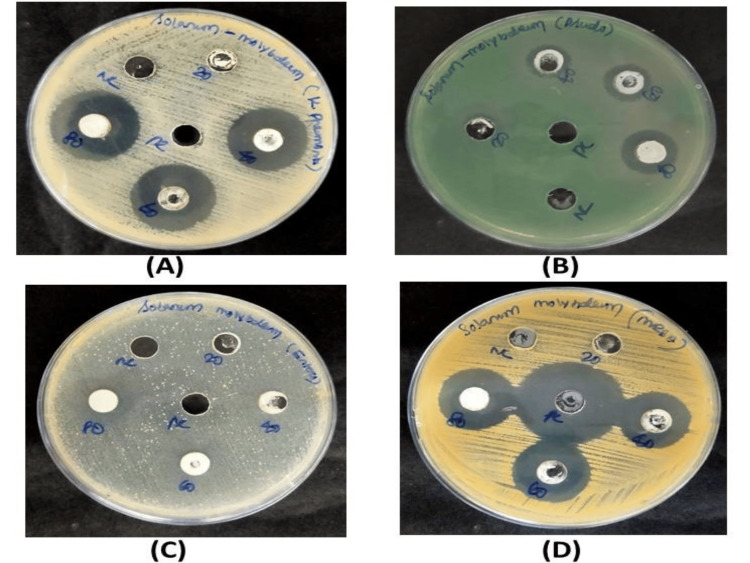
Antibacterial activity of synthesized Mo NPs using Solanum xanthocarpum against bacterial pathogens Klebsiella pneumoniae (A), Pseudomonas aeruginosa (B), Enterococcus faecalis (C), and methicillin-resistant Staphylococcus aureus (D) Mo NPs: Molybdenum nanoparticles

*Pseudomonas aeruginosa, Enterococcus faecalis, Klebsiella pneumoniae*, and methicillin-resistant *Staphylococcus aureus* were tested for the antibacterial activity of the produced Mo NPs using *Solanum xanthocarpum*. The inhibitory zone was determined according to that (Figure 7). As compared to the positive control (streptomycin), the 80 μl levels showed a 22 mm, 27 mm, 24 mm, and 24 mm inhibition against methicillin-resistant *Staphylococcus aureus, Klebsiella pneumoniae, Pseudomonas aeruginosa*, and *Enterococcus faecalis,* respectively.

Antioxidant activity of synthesized Mo NPs

**Figure 8:**
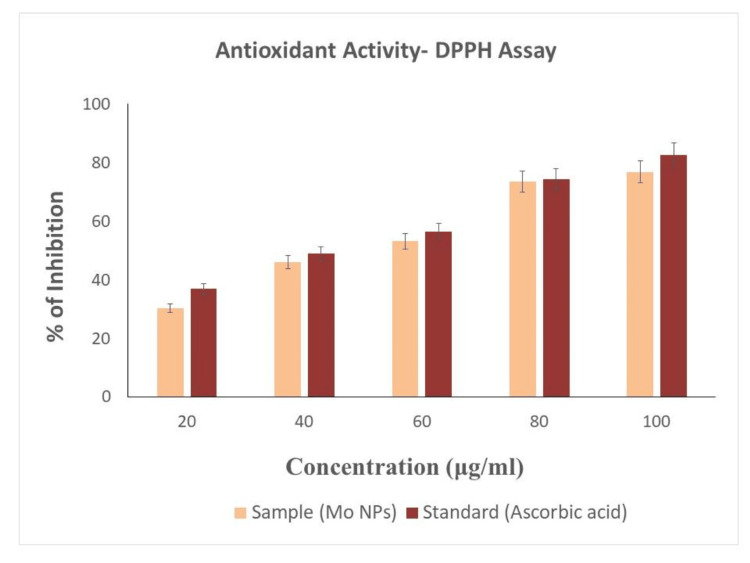
Antioxidant activity of synthesized Mo NPs using Solanum xanthocarpum DPPH: 2,2-diphenyl-1-picryhydrazyl; Mo NPs: Molybdenum nanoparticles

Figure 8 shows the antioxidant activity of the synthesized Mo NPs. The antioxidant activity was maximum at 100 μg/ml (73.49%), followed by moderate activity at 60 μg/ml (53.21%), and minimal activity at 20 μg/ml (30.21%), when compared to standard ascorbic acid.

## Discussion

In the current study, we used *Solanum xanthocarpum* as a reducing agent to produce Mo NPs. Surface plasmon resonance (SPR) is the cause of the nanoparticles' color change from dark brown to light brown. UV-Vis spectral analysis was used to determine the wavelength of the plasma resonance peak level of absorbance, and 320 nm was identified to be the peak level of absorbance.

FT-IR technique is employed to study the surface chemistry of metal nanoparticles that can be synthesized. It additionally allows for the existence of biomolecules and the chemical interactions that occur during the synthesis process between the different functional groups. Our synthesized Mo NPs using *Solanum xanthocarpum* were identified in the 500-4000 cm^-1^ wavelength range and values were 565.99 cm^-1^, 810.63 cm^-1^, 849.69 cm^-1^, 978.32 cm^-1^, and 2349.00 cm^-1^. Compounds included C-H, O=C=O, C-CI, and C=C, respectively. A similar study [16] revealed that Mo NPs using the *Canella Asiatica* plant were identified in the 400-4000 cm^-1^ range of wavelength. The peaks can be shown at 1015 cm^-1^, 1642 cm^-1^, 2846 cm^-1^, and 2926 cm^-1^. 

XRD method is used to identify if the related nanoparticles are crystalline or amorphous. Our synthesized Mo NPs mediated by *Solanum xanthocarpum* possessed a highly stable crystalline structure. The MoO_3_ XRD spectrum was compared to previous research, and it was found that the primary peaks at 12.7°, 25.6°, and 38.9° correspond to the MoO_3_ (020), (040), and (060) reflections, respectively. The crystalline structure of the MoO_3_ NPs and the extremely anisotropic α-MoO_3_ phase appear in the sharp peaks that have been observed [17]. According to another study [18], the XRD confirmed the photosynthesis of MoO_3 _NPs by determining the size of its crystals and analyzing the phase purity dimensions.

The size, shape, and distribution features of nanoparticles that confirm their presence were studied using SEM. The synthetic Mo NPs using *Solanum xanthocarpum* had an irregular shape, as shown by 1 μm and 0.5 μm magnifications. The agglomerated nanoparticles' average particle size was found to be around 200 to 300 nm. This study, compared with other studies, revealed that the Mo NPs using *Canella Asiatica* are agglomerated with an irregular morphology [16]. The elements present in *Solanum xanthocarpum *mediated Mo NPs were identified using EDX analysis. The Mo, O, and C signals were identified at 38.1, 40.4, and 21.5 keV, respectively. A previous study revealed that the Mo (64,04%) and O (35,96%) components were detected by weight in the EDX spectrum using synthesized MoO_3_ from *Nasturtium officinale* [18].

Antibacterial activity of the synthesized Mo NPs from *Solanum xanthocarpum *was tested using bacterial strains such as *Pseudomonas aeruginosa, Enterococcus faecalis, Klebsiella pneumoniae*, and methicillin-resistant *Staphylococcus aureus*. As compared to the positive control (streptomycin), the 80 μl levels showed a 22 mm, 27 mm, 24 mm, and 24 mm inhibition against methicillin-resistant *Staphylococcus aureus, Klebsiella pneumoniae, Pseudomonas aeruginosa*, and *Enterococcus faecalis, r*espectively. One previous study [17] examined the zone of inhibition for different concentrations of MoO_3_ and ampicillin (standard). The most significant zone of inhibition was found using 50 μl of MoO_3_ NPs and 50 μl of standard ampicillin. Using 50 μl of MoO_3_ NPs, the maximum zone of inhibition against *Klebsiella pneumonia* (20 ± 0:7) was shown. In contrast, a small zone of inhibition against *Staphylococcus aureus* (17 ± 0:3) and a moderate zone of inhibition against *Escherichia coli *and *Pseudomonas aeruginosa* were observed at the same dose. Furthermore, 50 μl of ampicillin exhibited a maximum zone of inhibition against *Escherichia* *coli* (22 ± 0:7) and *Pseudomonas aeruginosa* (22 ± 0:5), but an average zone of inhibition against *Staphylococcus aureus* (20 ± 0:1).

**Limitations** 

In the current study, we reported several types of in vitro analyses to evaluate the synthesized Mo NPs using *Solanum xanthocarpum*. Additional in vivo research, such as animal and clinical trials, will be helpful in better understanding its effects.

## Conclusions

We conclude that the environment-friendly synthesized Mo NPs from *Solanum xanthocarpum *exhibited antioxidant activity. Furthermore, the findings show that Mo NPs mediated by *Solanum xanthocarpum* can inhibit antibiotic-resistant bacteria, especially methicillin-resistant *Staphylococcus aureus, Klebsiella pneumoniae, Pseudomonas aeruginosa, *and *Enterococcus faecalis*. Furthermore, in vivo and clinical trials are needed to confirm the biocompatibility and bioefficacy of nanoparticles for most bacterial infections, notably MDR strains.
